# Surgical wait times and socioeconomic status in a public healthcare system: a retrospective analysis

**DOI:** 10.1186/s12913-022-07976-6

**Published:** 2022-04-29

**Authors:** Tyler J. Law, Derek Stephens, James G. Wright

**Affiliations:** 1 Center for Health Equity in Surgery and Anesthesia, University of California, San Francisco, USA; 2 Department of Anesthesia & Periopative Care, University of California, San Francisco, USA; 3 Zuckerberg San Francisco General Hospital, San Francisco, USA; 4 Department of Biostatistics, University of Toronto, Toronto, Canada; 5 The Hospital for Sick Children, Toronto, Canada; 6 Nuffield Department of Orthopedics, Rheumatology and Musculoskeletal Science, University of Oxford, Oxford, UK; 7 Ontario Medical Association, Toronto, Canada

**Keywords:** Surgery, Health equity, Socioeconomic status, Waiting times, Public health care

## Abstract

**Background:**

One aim of publicly-funded health care systems is to provide equitable access to care irrespective of ability to pay. At the same time, differences in socioeconomic status (SES) are associated with health outcomes and access to care, including waiting times for surgery. In public systems where both high- and low-SES patients use the same resources, low-SES patients may be adversely impacted in surgical waiting times. The purpose of this study was to determine whether a publicly-funded health system can provide equitable access to surgical care across socioeconomic status.

**Methods:**

Patient-level records were obtained from a comprehensive provincially-administered surgical wait time database, encompassing years 2006–2015 and 98% of Ontario hospitals. Patient SES was determined by linking postal code with the Material and Social Deprivation Index. Surgical waiting times (time in days between decision to treat and surgery) accounted for patient-initiated delays in treatment, and regression analysis considered age, SES, rurality, sex, priority level for surgical urgency (assigned by surgeons), surgical subspecialty, number of visits, and procedure year.

**Results:**

For the 4,253,305 surgical episodes, the mean wait time was 62.3 (SD 75.4) days. Repeated measures least squares regression analysis showed the least deprived SES quintile waited 3 days longer than the most deprived quintile. Wait times dropped in the initial study period but then increased. The proportion of procedures exceeding wait time access targets remained low at 11–13%.

**Conclusions:**

The least deprived SES quintile waited the longest, although the absolute difference was small. This study demonstrates that publicly-funded healthcare systems can provide equitable access to surgical care across SES.

## Introduction

Disparities in health care arise from many causes. One important cause is differences in socioeconomic status, which are associated with worse health outcomes and decreased access to care [1–3]. Patients with low-SES face challenges such as lack of transportation, child care, unavailability of caregivers for postoperative care, or receiving time off work [4–7]. This impeded access to care may result in major consequences to health and quality of life [8].

Evidence suggests that universal insurance coverage leads to more equitable access to care [9]. One important measure of surgical equity is waiting time for surgery. Existing research demonstrates that access as measured by waiting times is related to socioeconomic status (SES) [10]. An English study reported that surgical wait times and socioeconomic deprivation were correlated, while a Scottish study of cardiac surgery waiting times between 1986–1973 found longer wait times for the most deprived population [11, 12]. Though previous Canadian studies reported no association of surgical wait times with SES, all prior studies have focused on single disciplines, were performed in regions with a mix of privately- and publicly- funded health care, or did not adjust for patient-determined reasons for delay [13–17]. It remains an open question as to whether access to surgery is limited in a publicly-funded system.

In Ontario, Canada’s most populous province, surgical wait times are captured in a provincial database, providing a comprehensive record of wait times data. Surgical procedures (including all postoperative inpatient care) are funded as part of a public health insurance plan, and there is little out of province and no private care for medically necessary procedures [18, 19]. Thus, virtually all surgery takes place in the public health system, regardless of the patient’s ability to pay, making Ontario an ideal setting to evaluate surgical wait times for the most vulnerable members of society when the barrier of health insurance is removed.

The purpose of this study was to examine the association between SES and surgical waiting times in a publicly funded system using patient-level, population-wide surgical wait times data (inclusive of all procedures), from 2006–2015.

## Methods

### Wait times

Healthcare costs for medically indicated procedures for the 13 million Ontarians (including costs of surgery and hospitalization) are covered by a government single payer, the Ministry of Health and Long-Term Care. Little surgical care is provided outside the province.

The Ontario Wait Times (WTIS) database contains patient level wait times data from 98% of Ontario hospitals. Surgical wait times are calculated as the time from decision to treat until surgery. The database also contains demographic data, postal codes, and information related to the type of surgery. The study included all adult patients with complete data in the WTIS database from 2006–2015. We excluded patients < 18 years old at the time of surgery, missing wait time data, and missing or non-Ontario health card number.

Access targets for urgent and nonemergent care were previously established through expert consensus. Surgeons assign patients a priority 1 through 4 (P1-P4); with access targets ranging between 7–182 days [20–24]. Periods of delay due to “patient related” reasons, such as a change in medical status or patient rescheduling, are recorded as Days Affecting Readiness to Treatment (DART), and the calculated waiting time is adjusted for any DART days. During the study period, hospitals received extra targeted funds to increase surgical volume and reduce wait times for hip and knee replacements, cataracts, oncology procedures and cardiac surgery.

The following variables were obtained: Ontario health card number (unique individual identifier), birth year, sex, postal code, hospital, surgical specialty, procedure type, priority level, access target and DART reason.

### Socioeconomic status and rurality

The 2015 Postal Code Conversion File (PCCF, obtained from Statistics Canada) contains all the postal codes in Canada. Postal codes are combined by Statistics Canada into larger census groupings called Dissemination Areas (DAs). The PCCF also includes the relative rurality of each census subdivision. The Material and Social Deprivation Index (MSDI), developed by the Institut national de santé publique du Québec, uses Statistics Canada census data to calculate both material and social deprivation scores for each DA [25–27]. The MSDI separates DAs into deprivation quintiles containing 20% of Ontario’s population based on six indicators: average income, proportion of individuals without a high school diploma, proportion of employed individuals (material deprivation), and proportion of individuals living alone, proportion of lone parent families, and the proportion of separated, divorced or widowed individuals (social deprivation). We used the 2011 MSDI, the most recent version centered on the study period.

The PCCF was linked to the MSDI to obtain the material deprivation quintiles of each postal code in Ontario based on their DA. We merged the resulting database with the WTIS to link individual patients to their deprivation quintiles based on postal code. Rurality was classified as metropolitan areas (population at least 100,000), census agglomeration areas (CA) (two levels, each with population at least 10,000), or areas with strong to no metropolitan influence based on the proportion of commuting population. This measure has been previously used to examine correlations with health status [28].

The primary question was whether patients’ material deprivation levels were associated with surgical waiting times, controlling for the number of visits, age at procedure, material deprivation, rural status, sex, surgical specialty, priority level, and year of procedure. Social deprivation was found to have nearly the same distribution as material deprivation and so was not included in further analysis. Since patients underwent multiple procedures a fixed-effects, repeated measures least squares regression model using the Mixed Procedure in SAS was used. We used an Autoregressive AR(1) correlation matrix to model the correlation of the repeated waiting times. The correlation for this matrix decreases the further apart the waiting time within an individual. This structure made the most clinical sense. There was no concern with power of the study since the sample size was close to 4.5 million.

No individual patients were identified in this study. All data were presented with patient identifiers removed. The SAS software package V9.4 and Stata V15 was used for statistical analysis, and all data was analyzed by the authors. The research protocol was approved by the SickKids REB.

## Results

Four million, eight hundred and seventy-three thousand, two hundred and sixty-nine patient records containing wait times from 2006–2015 were obtained. 250,636 (5%) records were associated with a DA that had no deprivation data, which occurs when the community is too small, or a large proportion of the population lives in a collective dwelling (e.g. nursing home or prison). 369,328 pediatric cases were excluded, yielding 4,253,305 records for analysis.

A mean of 425,330 (SD 181,471) procedures were performed per year; 57% (2,412,557) of patients were female; with a mean age of 59.8 (SD 16.9); and 74% (3,148,728) of procedures were performed on patients from metropolitan areas. The mean waiting time for surgery was 62.3 (SD 75.4) days, with 95% of patients waiting 189 days or less.

On average, the least deprived quintile waited longest (mean 64.5 days, SD 78.5), and wait times decreased with each quintile (mean of most deprived group 60.7 days, SD 73.1) (Table 1). Median wait times were similar across quintiles (median 42–40 in least and most deprived, IQR 62 and 58). In analysis by geography, the most rural (no metropolitan influence) group waited longest on average (mean 71.8 days). The most deprived and most rural group had the longest waiting times in the cohort (mean 74.1 days, SD 109.7) (Table 1).

**Table 1:** Wait time in days, by deprivation quintile and metropolitan influence

**Metropolitan influence**	**Material Deprivation Quintile**											
	**Least deprived**		**2**		**3**		**4**		**Most deprived**		**Overall**	
	Mean (SD)	Med (IQR)	Mean (SD)	Med (IQR)	Mean (SD)	Med (IQR)	Mean (SD)	Med (IQR)	Mean (SD)	Med (IQR)	Mean (SD)	Med (IQR)
Metropolitan	65.2 (79.4)	42 (63)	63 (76.5)	41 (59)	62 (74.8)	41 (58)	61.2 (73.7)	40 (58)	60 (72)	39 (57)	62.4 (75.5)	41 (59)
CA area 1	54.7 (65.8)	36 (47)	57.2 (69.8)	37 (51)	59.5 (72.7)	39 (53)	58.3 (69.2)	39 (52)	57.5 (67.8)	37 (51)	57.6 (69.4)	38 (51)
CA area 2	61 (73)	40 (57)	60.5 (73.5)	39 (56)	62.5 (75.2)	40 (58)	60.9 (73.6)	38 (57)	62.6 (76.9)	39 (59)	61.5 (74.6)	39 (58)
Strong influence	62.6 (74.5)	42 (57)	63 (76.5)	41 (59)	64.6 (76.5)	42 (62)	60.2 (70.7)	40 (57)	63.3 (74.6)	42 (58)	62.8 (74.8)	41 (59)
Moderate influence	60.6 (75)	40 (59)	62.5 (74.7)	41 (59)	64.6 (74.3)	43 (61)	62.5 (74.2)	41 (58)	63 (73.6)	42 (59)	63 (74.2)	41 (59)
Weak influence	64.1 (75.5)	41 (63)	63.3 (76.4)	40 (60)	69.7 (95.2)	42 (67)	64.1 (82.2)	38 (62)	68.1 (88.3)	41 (66)	66 (84.5)	41 (64)
No influence	-	-	-	-	52.3 (152.9)	20 (52)	62.6 (68.9)	41 (59)	74.1 (109.7)	39 (70)	71.8 (111.2)	37 (68)
Overall	64.5 (78.5)	42 (62)	62.6 (75.9)	41 (59)	62.5 (75.5)	41 (59)	61.1 (73.5)	40 (58)	60.7 (73.1)	40 (57)	62.3 (75.4)	41 (58)

*CA* Census agglomeration

**Fig. 1:**
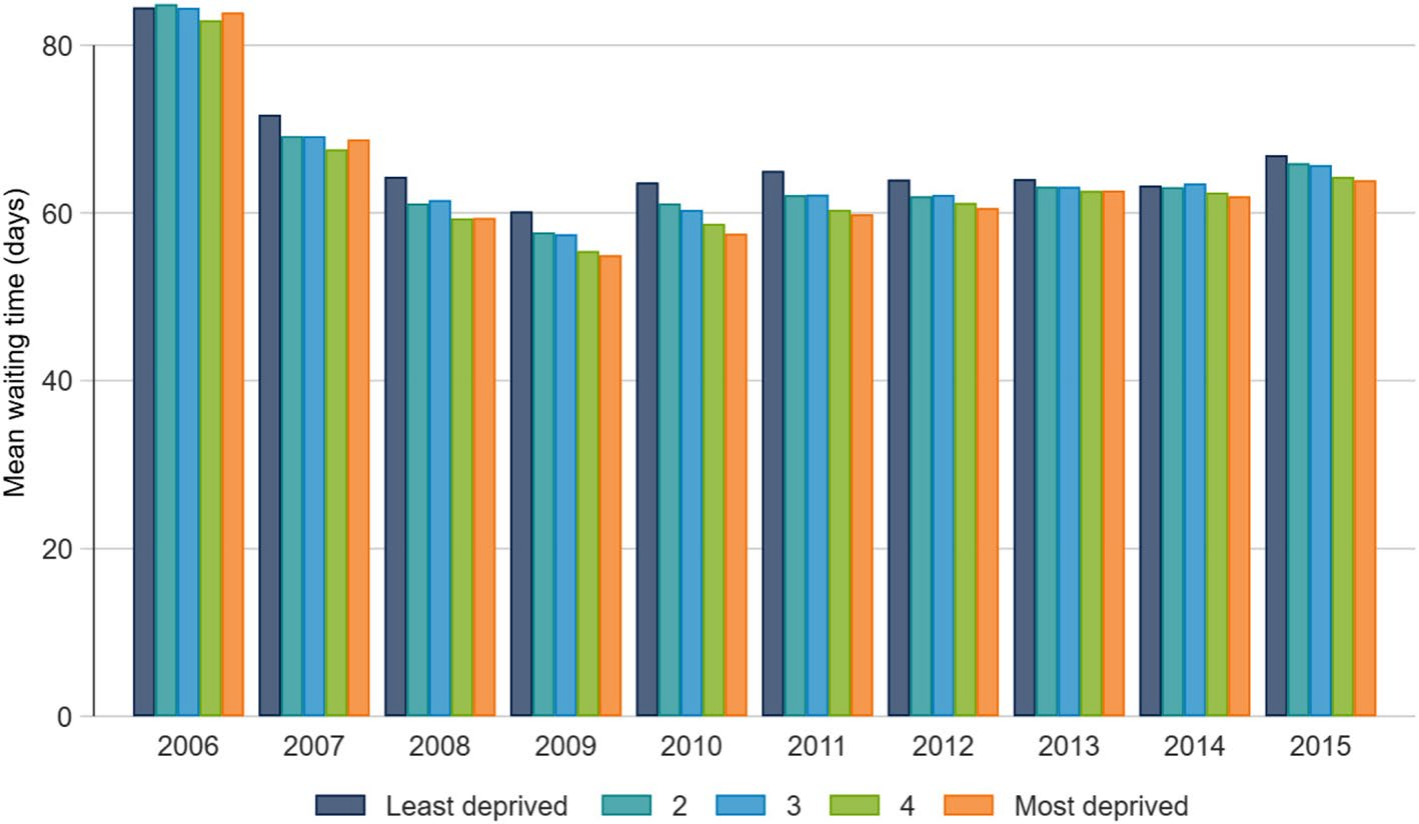
Mean waiting time according to material deprivation, by year

**Fig. 2:**
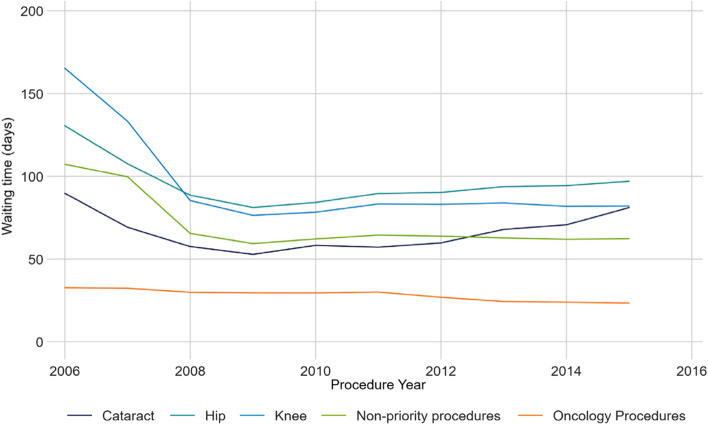
Mean waiting time of priority and non-priority procedures, by year

Mean wait times decreased in the initial period as hospitals began reporting waiting times. Since 2009, mean waits increased from 57.1 to 65.4 days (SD 71.0 to 74.8), with median waits undergoing a similar but smaller change (38 to 42, IQR 55 and 65 days) (Fig. 1). Fewer days accrued to the least deprived quintile (who wait a mean of 6.7 days longer than in 2009) than the most deprived quintile (who wait 9.0 days longer). Metropolitan areas experienced a mean 7.9-day increase in waiting times since 2009, while areas with no metropolitan influence experienced an increase of 20.0 days. Wait times for priority procedures (hips, knees, cataracts, oncology surgery) tended to increase since 2009, while waiting times for non-priority procedures decreased (Fig. 2). Median waits followed the same trends. The proportion of procedures exceeding access targets remained constant since 2009, ranging from 11–13%.

Regression analysis demonstrated a decrease in waiting times with each additional quintile of deprivation, such that the least deprived quintile waited 3.0 days longer than the most deprived quintile. Waiting times increased as metropolitan influence decreased, and areas with no metropolitan influence waited 14.8 days longer than metropolitan areas (Table 2).

**Table 2:** Adjusted difference in waiting times

	**Estimate**	**95% CI of estimate**	**Std. Err**	**p**
**Material Deprivation**				
Least deprived	3.0	2.7 to 3.2	0.1	< .0001
2	1.3	1.1 to 1.5	0.1	< .0001
3	1.3	1.1 to 1.5	0.1	< .0001
4	0.3	0.1 to 0.6	0.1	0.004
Most deprived	0	-	-	-
**Metropolitan influenced zone**				
1	-14.8	-18.0 to -11.6	1.6	< .0001
2	-19.6	-22.9 to -16.4	1.7	< .0001
3	-15.0	-18.3 to -11.8	1.7	< .0001
4	-14.6	-17.8 to -11.4	1.7	< .0001
5	-13.8	-17 to -10.5	1.7	< .0001
6	-10.1	-13.4 to -6.8	1.7	< .0001
No Metropolitan influence	0	-	-	-
**Gender**				
Female	3.1	3.0 to 3.3	0.1	< .0001
**Surgical subspecialty**				
General Surgery	2.6	2.0 to 3.3	0.3	< .0001
Gynaecologic Surgery	14.3	13.6 to 14.9	0.3	< .0001
Neurosurgery	17.3	16.4 to 18.2	0.5	< .0001
Oncology Procedures	-15.4	-16.0 to -14.7	0.3	< .0001
Ophthalmic Surgery	9.1	8.5 to 9.8	0.3	< .0001
Oral and Maxillofacial Surgery and Dentistry	28.9	28.1 to 29.8	0.4	< .0001
Orthopaedic Surgery	39.2	38.5 to 39.8	0.3	< .0001
Otolaryngic Surgery	36	35.3 to 36.7	0.4	< .0001
Plastic and Reconstructive Surgery	14.8	14.1 to 15.5	0.4	< .0001
Thoracic Surgery	1.3	-0.2 to 2.9	0.8	0.09
Urologic Surgery	-3.9	-4.6 to -3.2	0.3	< .0001
Vascular Surgery	0	-	-	-
**Priority Level**				
1	-65.8	-66.6 to -65.1	0.4	< .0001
2	-42.6	-43.0 to -42.3	0.2	< .0001
3	-19.2	-19.4 to -19.1	0.1	< .0001
4	0	-	-	-
**Age at procedure**	**0.1**	**-**	**0.003**	** < .0001**
**Procedure year**	**-0.12**	**-**	**0.02**	** < .0001**

Estimate represents difference in days compared to reference group. Reference groups: Deprivation—Most deprived quintile, MIZ—No metropolitan influence areas, priority level—priority 4, Sex—Male, Surgical subspecialty—Vascular Surgery

**Fig. 3:**
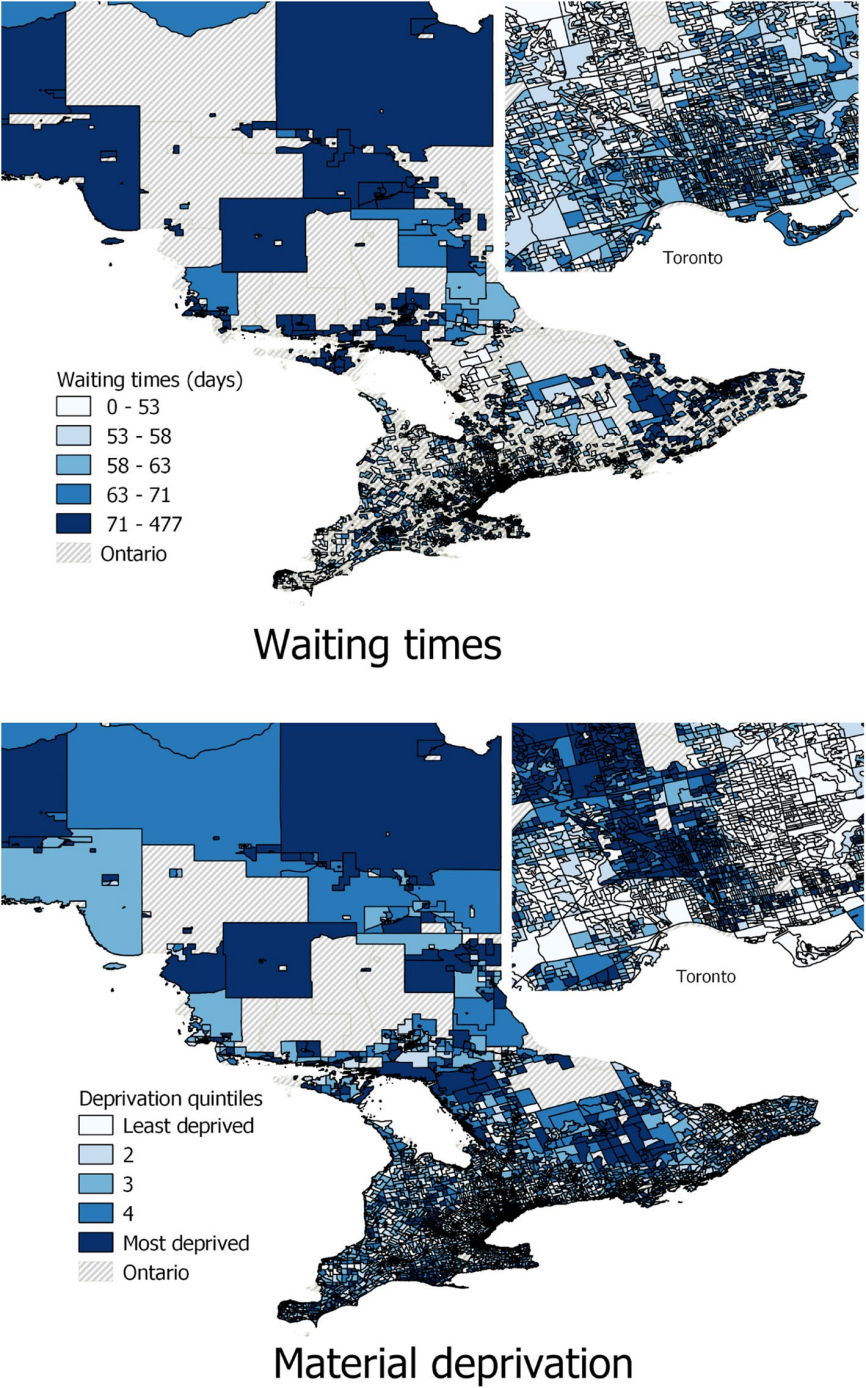
Geographic distribution of waiting time and material deprivation

Female gender was associated with a 3.1 day increase in waiting time compared to male gender. Analysis by surgical speciality demonstrated a wide range of adjusted waiting times, with a range of 54.6 days between orthopedic and oncology surgery (oncology procedures from all specialties are categorized together in the database) (Table 2). Mapping of the data by dissemination area demonstrates the lack of correlation between the geographic distribution of SES and the distribution of waiting times (Fig. 3).

## Discussion

Wait times have been identified as an important access to care issue [29]. Significant resources have been invested in Ontario towards reducing wait times as a key measure of quality of care. A 10-year Canadian federal strategy was initiated in 2004, with additional federal and provincial funding allocated to wait times [30, 31]. In this study performed in a publicly-funded system without private market for medically-indicated care, surgical waiting times were not related to socioeconomic status. After adjustment, the least deprived quintile waited slightly longer than other quintiles overall, although the differences were not clinically important. A larger difference in adjusted analysis was found across geography, with the most rural areas waiting up to 20 days longer than the area with the shortest waiting time (Census agglomeration area 2). However, while this is statistically significant and may represent an important difference for the most rural areas, this fraction of the Ontario population comprised only 2,139 cases, (0.05%). Thus, in Ontario the most vulnerable patients waited no longer for their surgery, confirming that access to surgical care was equitable for those of low SES. This is particularly timely information as COVID-19 has had deleterious effects on access to elective surgery, and the impact of delayed elective procedures may lead to significant strain on systems and patients [32–35].

Wait times in Ontario decreased significantly at the introduction of the WTIS and additional targeted funding. This may reflect an immediate impact on backlog, but may also be because not all specialties were included until 2009. Procedures identified as a priority experienced an increase in wait times over the study period, while non-priority procedures decreased. This suggests that increased focus did not redirect resources away from the non-priority procedures, but does raise questions as to the effectiveness of the targeted funding. The gradual increase in wait times later in the study period may reflect the uncovering of unmet surgical need [36].

Area-based metrics of social and material status may differ from the individuals’ realities. Various versions of the MSDI have undergone robust validation and have performed favourably. A survival study comparing individual to area-based inequity showed that the MSDI showed directionally similar hazard-ratios of survival, but of a smaller magnitude than when calculated individually, particularly among the more deprived groups. These differences were more pronounced in more rural geographies [37]. A study done assessing the MSDI along multiple axes of validity noted that the index performed well in these areas [38]. Health inequalities are independently related to both geographic and individual metrics, reflecting that individual experience is influenced by both [39].

Important strengths of this study were the unique combination of a constrained population and single payer system that captures virtually all surgery done in the province. Second, the data allowed adjustment for important SES confounders, including a measure of rurality. Third, procedures had a priority coding, allowing adjustment for emergent and non-emergent procedures. Fourth, the use of DART days in the calculation of waiting times accounted for when patients decided to delay surgery, which no prior study has included.

There are several potential limitations of this study. We excluded patients without a postal code, so those with severe material deprivation (e.g. those with no home) would not be captured. However, this likely represents an extremely small fraction of the population. Second, surgeon assigned priorities are subject to gaming, as surgeons may inflate priorities to advocate for more resources, or assign access targets that they feel confident can be met. However, this would not affect mean waiting times. Third, there may be important delays in the time between surgical referral and actual appointment date that could be affected by SES. The surgical system may be equitable for those booked for surgery, but inequitable in access to specialist appointments themselves. Data for this type of delay was not available at the time of the study, but is being incorporated into the WTIS.

In conclusion, this study demonstrates that in a publicly-funded healthcare system, surgical waiting times for patients are not related to SES. However, mean waiting times increased during the study period, despite concerted government efforts otherwise.

## Data Availability

The data that support the findings of this study are available from Cancer Care Ontario but restrictions apply to the availability of these data, which were used under license for the current study, and so are not publicly available. Data are however available from the authors upon reasonable request and with permission of Cancer Care Ontario.

## Abbreviations

SES: Socioeconomic status
WTIS: Wait Times Information System
DART: Days Affecting Readiness to Treatment
PCCF: Postal Code Conversion File
DA: Dissemination Area
MSDI: Material and Social Deprivation Index
